# Atomic-scale identification of Pd leaching in nanoparticle catalyzed C–C coupling: effects of particle surface disorder†
†Electronic supplementary information (ESI) available: Materials and methods, UV-vis spectra, raw EXAFS data, and computational methods. See DOI: 10.1039/c5sc01424g


**DOI:** 10.1039/c5sc01424g

**Published:** 2015-07-23

**Authors:** Beverly D. Briggs, Nicholas M. Bedford, Soenke Seifert, Hilmar Koerner, Hadi Ramezani-Dakhel, Hendrik Heinz, Rajesh R. Naik, Anatoly I. Frenkel, Marc R. Knecht

**Affiliations:** a Department of Chemistry , University of Miami , 1301 Memorial Drive , Coral Gables , Florida 33146 , USA . Email: knecht@miami.edu; b Materials and Manufacturing Directorate , Air Force Research Laboratory , Wright-Patterson Air Force Base , Ohio 45433 , USA; c Applied Chemicals and Materials Division , National Institute of Standards and Technology , 325 Broadway , Boulder , Colorado 80305 , USA; d X-Ray Science Division , Argonne National Laboratory , 9700 S. Cass Ave , Argonne , Illinois 60439 , USA; e Department of Polymer Engineering , University of Akron , Akron , Ohio 44325 , USA; f Department of Physics , Yeshiva University , 245 Lexington Ave , New York , New York 10016 , USA

## Abstract

C–C coupling reactions are of great importance in the synthesis of numerous organic compounds, where Pd nanoparticle catalyzed systems represent new materials to efficiently drive these reactions. Despite their pervasive utility, the catalytic mechanism of these particle-based reactions remains highly contested. Herein we present evidence of an atom leaching mechanism for Stille coupling under aqueous conditions using peptide-capped Pd nanoparticles. EXAFS analysis revealed Pd coordination changes in the nanoparticle consistent with Pd atom abstraction, where sizing analysis by SAXS confirmed particle size changes associated with a leaching process. It is likely that recently discovered highly disordered surface Pd atoms are the favored catalytic active sites and are leached during oxidative addition, resulting in smaller particles. Probing the mechanism of nanoparticle-driven C–C coupling reactions through structural analyses provides fundamental information concerning these active sites and their reactivity at the atomic-scale, which can be used to improve catalytic performance to meet important sustainability goals.

## Abstract 2

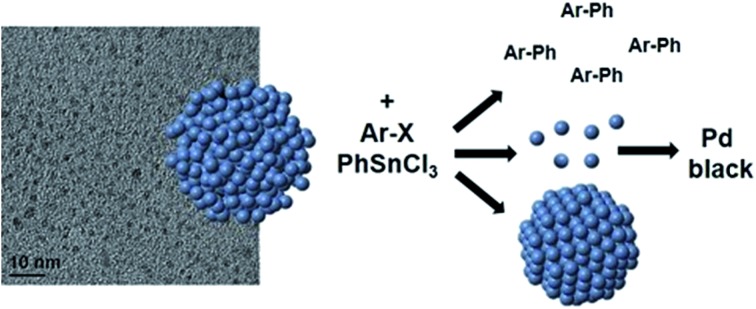
Atomically-resolved X-ray-based methods demonstrate that Pd atoms are leached *via* oxidative addition during nanoparticle-catalyzed Stille coupling under ambient reaction conditions.

## Introduction

Carbon–carbon (C–C) coupling reactions are pervasively used for chemical synthesis; however, they typically require unsustainable conditions of high temperatures, organic solvents, and high Pd loadings.^1^,^2^ Conversely, Pd nanoparticles (NPs) could advance these reactions toward more sustainable conditions, where such materials have recently been employed to drive Stille, Suzuki, and Heck couplings under favorable conditions such as ambient temperatures, aqueous solvents, and ultra-low catalyst concentrations.^1^,^3^–^6^ Unfortunately, the mechanism by which Pd NPs drive these processes remains unclear and greatly contested. By understanding catalytic mechanisms at the atomic level, rationally designed catalysts with increased reactivity under sustainable conditions could be developed.

It is generally accepted that C–C couplings follow a three-step process: oxidative addition, transmetalation, and reductive elimination.^7^ For oxidative addition, NP-driven systems have been proposed to operate by two different possible approaches: a surface or metal atom leaching mechanism.^1^,^4^,^8^ In the surface mechanism, the actual coupling process occurs directly at the metallic interface; however, for the leaching process, oxidative addition results in the abstraction of active Pd species from the NP to catalyze C–C bond formation in solution. To advance these materials for enhanced activity, it is imperative that the exact mechanism be determined, especially under ideal conditions of aqueous solvents at room temperature to achieve sustainable reactivity where minimal information is known.

There are several compelling arguments to support either a surface-based^9^–^17^ or atom-leaching mechanism.^18^–^22^ Work by Ellis *et al.* involving an in-depth spectroscopic study of Suzuki coupling suggested that the mechanism operated on the Pd NP surface.^9^ Using X-ray methods, normalization of the reaction turnover frequency (TOF) indicated that the reaction occurred directly on the NP surface at edge and vertex atoms.^9^ Continuation of these studies with further *in operando* X-ray experiments, as well as spiking and Hg poisoning studies, lended additional support to a surface-based process for Suzuki coupling.^12^ Note that these reactions were processed in toluene at 60 °C. Furthermore, work done by Lambert and coworkers has indicated that Sonogashira cross coupling can occur solely on the surface of Ag^13^ and Au;^14^,^15^ however, these studies used single crystalline metallic surfaces and not NPs that may possess disordered surface metal atoms that could affect the reactivity. In addition, studies exploring a surface mechanism for Suzuki coupling were performed by Wang *et al.* where Pd NPs were deposited on Au nanorods. In this approach, the plasmonic properties of the Au component enhanced the reactivity of the Pd materials.^16^,^17^ Through normalization by the number of surface atoms of Pd, it was postulated that the coupling process was occurring directly on the Pd surface.^16^

Conversely, extensive studies have indicated that the C–C coupling process does not occur on the Pd NP surface, but by Pd atoms abstracted from the NP in solution.^18^–^22^ For instance, work by Li and coworkers that employed poorly crystalline Pd NPs demonstrated that the materials increased their structural order after the reaction, suggesting that defect (*i.e.* disordered) surface Pd atoms were leached during the coupling process.^18^ Other work also supports a leaching mechanism for C–C coupling, such as Stille TOF studies based upon Pd NP loading^19^ as well as studies from Reetz and Westermann^21^ and de Vries^22^ that detail the *in situ* formation of NPs that are the responsible species for catalysis. Such Pd NP formation was shown where periodic TEM samples taken during Heck coupling using a Pd precatalyst revealed the formation of 1.6 nm NPs.^21^ Interestingly, product formation was not noted until NPs were observed, suggesting that these materials were the active species.^21^ Further work on ligand-free Pd catalysts in the Heck reaction supported the generation of NPs in C–C coupling systems that employ Pd leaching during oxidative addition.^22^ Finally, theoretical studies also have shown that atom abstraction can be the rate-determining step, even if Pd atoms are removed only a short distance from the surface (3 Å or more).^23^,^24^ Taken from all of these contrasting studies, it is evident that further work is necessary to elucidate the mechanism of NP-driven C–C coupling.

To achieve this mechanistic understanding, it is important that the individual steps of the reaction mechanism be fully explored in isolation. Most previous studies have examined the C–C coupling process as a whole, rather than speciating each step. Such studies are necessary, especially of the initial oxidative addition step, to determine their effects on the particle structure, which could reveal changes associated with the reaction (supporting leaching) or the lack of any structural change (supporting a surface-based process). Should the C–C coupling process be sufficiently fast, Pd NP structural changes may not be observed, necessitating that each step be studied individually. In addition, most studies employ unsustainable conditions of high temperatures and organic solvents, where an understanding of the effects of the reaction mechanism on the NP under energy neutral (room temperature) and aqueous conditions is required to advance their reactivity.

In this study, we have employed Extended X-ray Absorption Fine Structure Spectroscopy (EXAFS), in combination with Small Angle X-ray Scattering (SAXS), to examine the Stille coupling reaction catalyzed by peptide-capped Pd NPs under room temperature and aqueous conditions. Such materials have been shown to be reactive where previous studies suggested that a Pd atom leaching process may be occurring;^23^ however, no experimental correlation between particle structural changes and the reaction mechanism are known. Such effects could likely be translated to other systems under similar conditions, thus expanding the fundamental understanding of Pd NP-driven C–C coupling for enhancement to achieve optimal and long–term reaction efficiency. Based upon changes in the Pd–Pd coordination numbers (CNs) and overall NP dimensions as a function of the reaction progression, the results strongly support a Pd atom leaching process occurring during oxidative addition under the selected conditions. In the reaction, disordered surface Pd atoms^23^ on the NP are abstracted, leaving behind a more ordered core. Once leached, the coupling ensues in solution, wherein the liberated Pd species continue to cycle through the reaction. As such, the particle is speciated into two regions: highly reactive disordered surface atoms that are the favored catalytic species and core atoms with lower reactivity. This work presents important new findings on the reactivity of NPs that could play a significant role in the design of new materials for increased reactivity under sustainable conditions.

## Results and discussion

### Pd NP synthesis

**Fig. 1:**
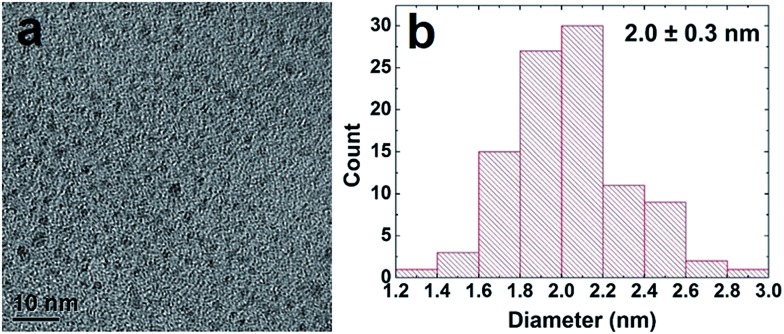
TEM characterization of the peptide-capped Pd NPs. Part (a) presents the TEM image, while part (b) displays the particle sizing analysis.

Pd NPs were prepared using the Pd-specific Pd4 peptide (TSNAVHPTLRHL) based on described methods.^5^ UV-vis analysis (ESI, Fig. S1†) indicated the emergence of a ligand-to-metal charge transfer band at 224 nm in a solution of Pd^2+^ and Pd4, prior to BH_4_^–^ addition, consistent with the Pd^2+^ coordinating to the peptide amines.^5^ After reduction, the shoulder disappeared and the resulting spectrum was characteristic of NP formation. Transmission electron microscopy (TEM) analysis, shown in Fig. 1a, confirmed the formation of quasi-spherical and nearly monodisperse Pd NPs. As indicated by the particle sizing analysis (Fig. 1b), the peptide-capped NPs have an average diameter of 2.0 nm ± 0.3 nm, consistent with previous studies.^5^

### Isolation of the oxidative addition step

**Fig. 2:**
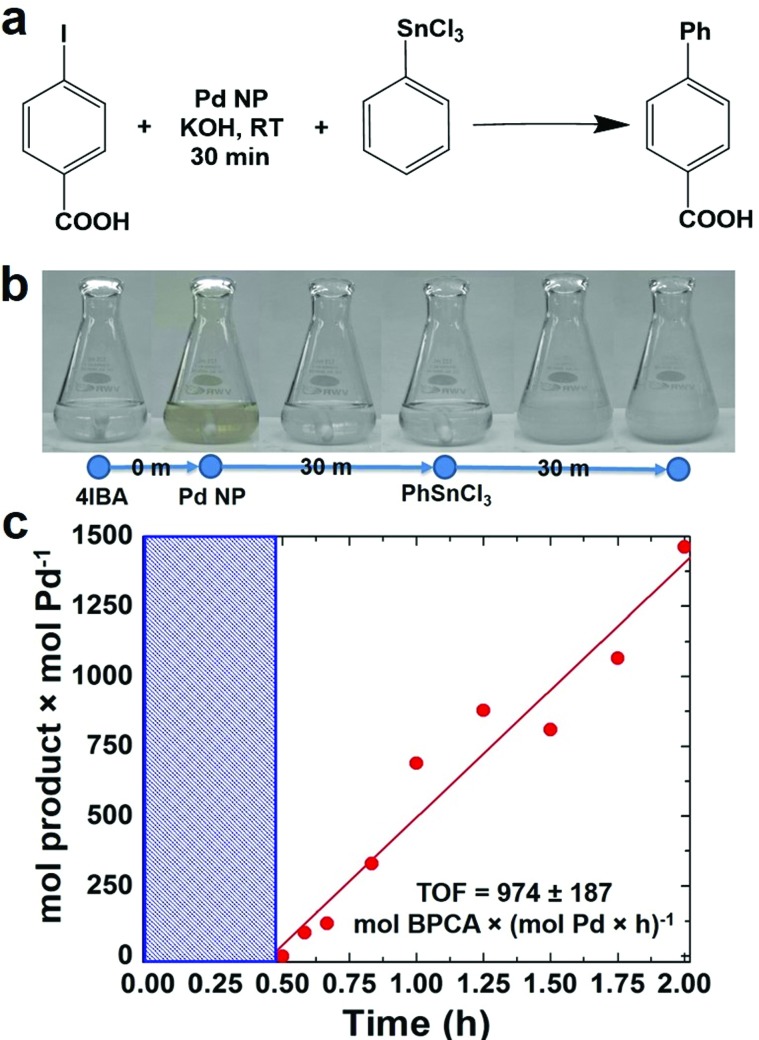
Isolation of the oxidative addition step using Pd4-capped Pd NPs at 0.05 mol% Pd. Part (a) presents the overall reaction, while part (b) displays the color change during oxidative addition. Finally, part (c) shows a TOF reaction analysis plot.

In an initial study, the NPs were employed to drive a modified Stille coupling reaction (Fig. 2a), focused specifically on the oxidative addition step for Pd leaching. Typically when the peptide-capped materials are used, the aryl halide, 4-iodobenzoic acid (4-IBA) is commixed with the transmetalation reagent, PhSnCl_3_, followed by NP addition to drive the reaction.^25^ Under these standard conditions, the entire reaction proceeds to product formation.^5^ Furthermore, due to the fast reactivity, it is nearly impossible to separate oxidative addition to elucidate the possibility of metal atom leaching. In the modified system, the NPs were added to a solution of 4-IBA at a Pd loading of 0.05 mol%, in the absence of PhSnCl_3_. This allows for oxidative addition to occur without continuing on to transmetalation. Initially, the characteristic brown color of Pd NPs was observed at the start of the reaction (Fig. 2b). The color intensity diminished and, within 30 min, the solution became clear and colorless, suggesting that the NPs were no longer present. Next, PhSnCl_3_ was added to initiate transmetalation. Within 15 min, a white precipitate was observed, corresponding to the formation of the product, biphenylcarboxylic acid. Using the addition of the PhSnCl_3_ as the reaction starting point, aliquots were taken and the product was quantified to calculate the TOF value (Fig. 2c). Under these modified conditions, a TOF of 977 ± 187 mol product (mol Pd × h)^–1^ was observed. Note that this value is diminished when compared to the standard reaction that does not isolate the oxidative addition step.^26^ These diminished TOF results are not surprising; should the Pd NPs be completely leached to Pd^2+^ complexes in solution, a higher concentration of regenerated Pd^0^ species after the reaction would be developed. At these high Pd^0^ concentrations, aggregation to form Pd black would be anticipated. Since Pd black is comparatively unreactive, this would lead to the observed reduction in catalytic activity.

From this analysis, it is evident that a change in NP morphology occurred during oxidative addition due to the color change. While this analysis is able to isolate oxidative addition, it cannot identify what changes to the NPs actually occurred, which is quite challenging; due to their low solution concentration, observation of a statistically relevant population of NPs after the reaction *via* TEM is not possible. As a result, spectroscopic methods are required.

### EXAFS studies

To fully elucidate structural changes to the Pd NPs as a function of the reaction, spectroscopic methods must be employed. Such approaches are required due to the low concentrations that prohibit NP observation *via* TEM after the reaction. EXAFS studies of the materials were conducted for various steps of the Stille coupling process, including isolation of oxidative addition. Pd NP solutions were prepared and transferred to a liquid cell for each analysis, where the materials were examined under three specific conditions: in the alkaline solvent before the reaction (termed Pd NP), in the presence of the aryl halide only (termed Pd NP + 4IBA; *i.e.* the modified reaction of Fig. 2 to isolate oxidative addition from the whole reaction), and after the Stille coupling process was complete (termed post reaction). For all studies of the materials after the reaction throughout the text, the system was processed wherein the oxidative addition step was not isolated first. In this regard, the 4-IBA and PhSnCl_3_ were commixed, to which the Pd NPs were then added to drive the reaction. Since the reaction rate of the system is exceedingly fast, *in situ* analysis of the process *via* EXAFS was not possible. The aligned, averaged, background-subtracted, and edge-step normalized spectra were fit in *r*-space using FEFF6 theory^27^,^28^ to determine the local Pd environment.

**Fig. 3:**
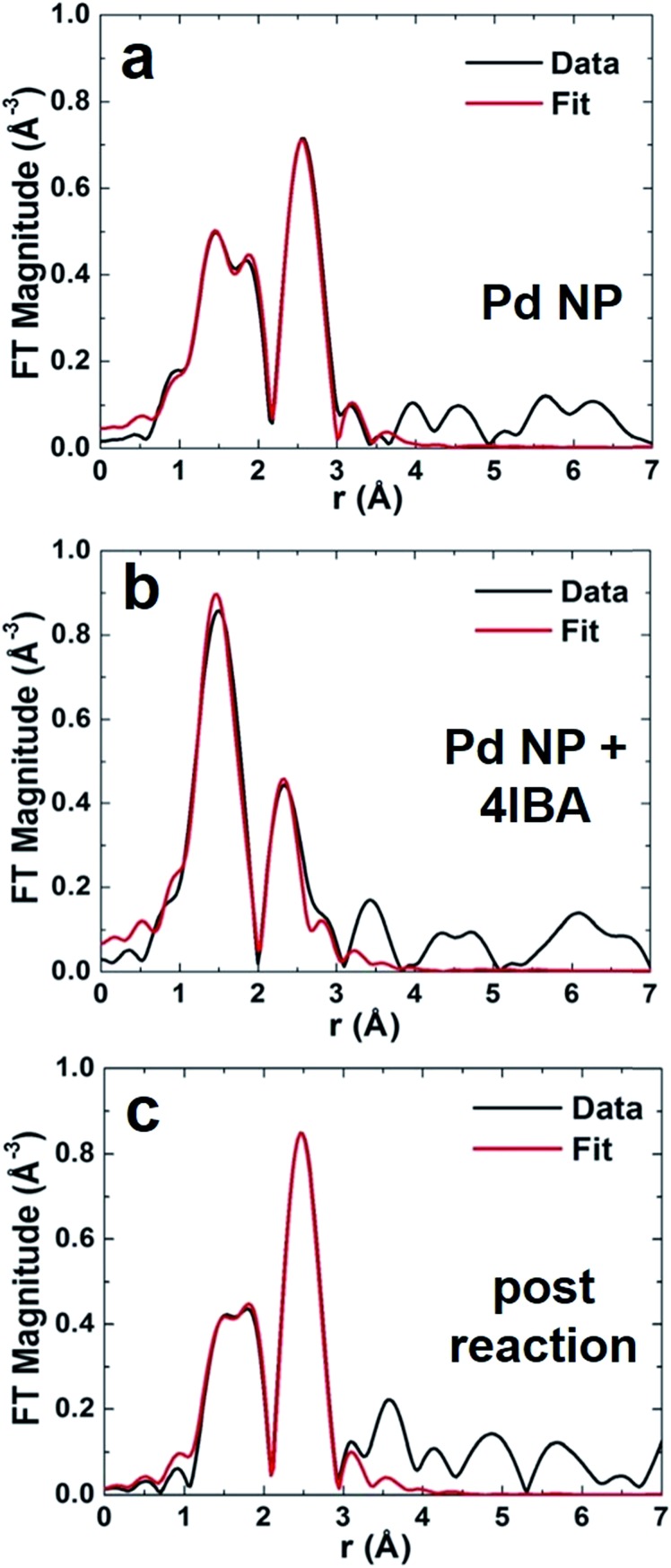
EXAFS *r*-space analysis at the Pd *k*-edge for (a) Pd NPs before the reaction, (b) Pd NP + 4IBA that isolates oxidative addition, and (c) the materials after Stille coupling completion. The *k*-ranges for Fourier transforms of *k*^2^-weighted EXAFS data were: (a) 2–10 Å^–1^, (b) 2–10 Å^–1^, and (c) 2–9.9 Å^–1^.

The *r*-space data (black plots) with corresponding fits (red plots) are shown in Fig. 3. Initially, the Pd NPs were examined in 1.125 M aqueous KOH, the reaction solvent, in the absence of any Stille coupling reagents (Fig. 3a). Note that this is not a catalytic process, but examines the stability of the particles in the reaction medium. In the *r*-space data, two peaks (not phase-corrected) are clearly evident: one between 2 and 3 Å, corresponding to the Pd–Pd first coordination shell, and a second one between 1.5 and 2.2 Å, due to the Pd–C/O/N contribution. This second peak arises from the peptides bound to the Pd, where EXAFS cannot distinguish between C, O, and N due to their similar scattering profiles. When the Pd NPs were then examined in the same medium, but in the presence of 4-IBA, dramatic changes in the *r*-space data was evident (Fig. 3b). For this system, the oxidative addition process is isolated from the overall Stille coupling process to clearly observe the potential effects of Pd leaching using the 4-IBA substrate. In this system, the peak corresponding to the Pd–C/O/N interaction remained; however, the second peak shifted to a lower *r*-range, demonstrating that no Pd–Pd bonds remained (Fig. 3b). It is likely that the second peak originates from Pd–I interactions, as discussed below. The lack of a visible Pd–Pd contribution in this EXAFS spectrum suggests that the NPs have been decomposed, consistent with the observed solution color change of Fig. 2 and a Pd leaching process. The last analysis focused on the Pd NPs after the Stille coupling reaction was complete (Fig. 3c). For this system, both the 4-IBA and PhSnCl_3_ were commixed prior to Pd NP addition, thus the reaction would immediately go to completion without isolation of the oxidative addition step. Note that the reaction was finished within 5 min, which is too short to analyze *via in situ* EXAFS at the Pd concentrations used here. This sample again displayed a peak from Pd–C/O/N contributions, and a second peak consistent with Pd–Pd bonds. These tentative attributions of the EXAFS peaks to specific pair interactions in real space were used to guide theoretical modeling, as discussed below.

From the EXAFS analysis, first nearest neighbor CNs were determined to elucidate NP structural information (Table 1). For the Pd NPs in KOH, Pd–Pd and Pd–C/O/N CNs of 3.6 ± 0.8 and 1.8 ± 0.6, respectively, were noted, consistent with previous analysis of Pd4-capped Pd NPs in the solid state.^25^ This indicates that the alkaline environment does not alter the structure, thus any changes to the materials arise from the Stille reaction. Interestingly, the Pd–Pd CNs are lower than expected; for a 2.0 nm Pd NP, a CN of ∼9 is anticipated.^29^ The present value is likely diminished for two reasons: incomplete reduction of Pd^2+^ and undercoordination of highly disordered surface Pd atoms.^23^ Estimation of the CN values for the peptide-capped Pd NPs can be ascertained from previously published NP configurations elucidated *via* pair distribution function analysis.^23^ This study elucidated the complete NP structure, including both the significant surface metal atom disorder and the orientation of the peptides on the NP interface. Nearest neighbor analysis using atomistic models of the peptide-covered particle by molecular dynamics simulations at room temperature indicates average Pd–Pd CN values of 5.8 ± 0.2 as well as a Pd–C/O/N CN of 0.5 ± 0.1 (see ESI† for details of the calculation). Note that these values are for the NPs alone without the unreduced Pd^2+^ complex in solution. Even with this, the estimation still remains below the anticipated value, which occurs due to significantly undercoordinated Pd^0^ atoms on the NP interface. When the Pd NPs were studied in the presence of 4-IBA that isolated the oxidative addition step from the complete reaction, no Pd–Pd interactions were evident; both Pd–I and Pd–C/O/N contributions were used to fit the data. To this end, a Pd–I CN of 1.0 ± 0.5 was obtained, while a Pd–C/O/N CN of 2.5 ± 0.5 was noted. For the materials studied after the Stille reaction was compete, a CN change was evident. Here a Pd–Pd and Pd–C/O/N CN of 5.1 ± 1.1 and 0.8 ± 0.4 were calculated, respectively. Note that other theoretical EXAFS contributions were attempted for each sample, but all resulted in a much worse fit.

**Table 1:** CNs and bond lengths from EXAFS analysis

Experiment	Bond	CN	Bond length (Å)
Pd NP	Pd–Pd	3.6 ± 0.8 (5.8^*a*^)	2.74 ± 0.01
	Pd–C/O/N	1.8 ± 0.6	2.01 ± 0.03
Pd NP + 4IBA	Pd–Pd	not present	not present
	Pd–C/O/N	2.5 ± 0.5	2.00 ± 0.01
	Pd–I	1.0 ± 0.5	2.60 ± 0.01
Post reaction	Pd–Pd	5.1 ± 1.1	2.71 ± 0.01
	Pd–C/O/N	0.8 ± 0.4	2.00 ± 0.04
	Pd–I	not present	not present

^*a*^Theoretical CN determined from modelling of Pd4-capped Pd NPs.

Taken together, the EXAFS results support a significant structural change in the NPs during oxidative addition. Such a change is consistent with Pd leaching during oxidative addition, forming a Pd^2+^ intermediate in solution. Under the conditions used, both to isolate oxidative addition and drive the full reaction, sufficient 4-IBA is present to fully oxidize all of the Pd in solution; however, when considering the complete reaction, PhSnCl_3_ is present in the reaction mixture for transmetalation to occur, driving the reaction forward to completion and quantitative product formation. Note that no byproducts or homocoupling was observed in the reaction, confirming that oxidative addition occurred, rather than a secondary process leading to Pd NP degradation.

Overall, the EXAFS results support the leaching mechanism for Stille coupling driven *via* oxidative addition. In this regard, the Pd4-capped Pd NPs were noted to be stable in the basic medium. This indicates that any changes to the material structure are directly related to the catalytic process and not the conditions. Taking into account the considerations above, a Pd–Pd CN value of 5.8 was computationally derived using atomistic models for these materials prior to the reaction. When the NPs are exposed to the aryl halide only in the absence of PhSnCl_3_ (*i.e.* isolation of the oxidative addition step), no Pd–Pd bonds are observed; however, increased Pd–C/O/N bonds and a new Pd–I bond with a CN of ∼1 are noted. Such effects are consistent with oxidative addition abstracting Pd^2+^ to solution. Should the NP remain intact with oxidative addition occurring at the metal interface, the complete loss of Pd–Pd bonds would not be observed. Additionally, the increase in the Pd–C/O/N CN could come from the coordination of the phenyl ring to the Pd^2+^, which cannot be easily distinguished from the peptide. Note that the sum of the CN environment around these Pd species is ∼3.5, which is very close to the anticipated value of 4 for such complexes.

When the NPs were examined *via* EXAFS after the complete Stille reaction was processed, an interesting change in the bond lengths was noted. The bond length significantly contracted from 2.74 Å to 2.71 Å (Table 1). A contraction in the bond length supports the existence of smaller particles in the reaction medium after the Stille coupling process reached completion.^30^ Such results are also directly supported by SAXS studies of the same materials (described below). It has been established that as NP size decreases, the metal–metal bond length decreases for very small materials due to surface strain.^31^,^32^ These changes likely arise from the effects of the Stille reaction on the NPs. As recently indicated, the highly disordered atoms at the NP surface are easily abstracted.^23^,^24^ Should only these disordered surface atoms be leached, a more ordered core of a smaller size would remain, giving rise to an overall decrease in the Pd–Pd bond length due to the contracted NP diameter. Note that precipitation of the Stille product occurs, which likely removes the unreacted Pd^2+^ complex from the solution and the analysis. This is evident due to the dramatic decrease in the Pd–C/O/N CN value for the NPs after the reaction. Additionally, the leached Pd atoms may aggregate to bulk Pd black upon reaction completion, where a gray precipitate was noted. These precipitates were not likely to be observed *via* EXAFS as they fell outside the X-ray beam; however, a small fraction may be included, slightly contributing to the Pd–Pd CN. Overall, the EXAFS analysis supports Pd abstraction occurring at the NP interface, where the highly disordered species are leached and the more ordered core remain. Once the disordered atoms are leached, the reaction ensues from these species; leaching of Pd from the more ordered core is not likely to be favored as compared to oxidative addition at the pre-leached Pd atoms in solution. As such, the core species are potentially less reactive.

While the data supports a Pd atom leaching process, it cannot rule out the abstraction of small Pd clusters as the reactive species in solution. Unfortunately due to the speed of the reaction, it is not possible to monitor the abstraction process *via* EXAFS *in situ*, thus the exact identity of the reactive species cannot be quantitatively defined. However recent studies on the calculated abstraction energies of Pd atoms on the surface of the disordered peptide-capped Pd NP suggest that the leaching process occurs for single atoms at the NP surface.^23^

### SAXS studies

**Fig. 4:**
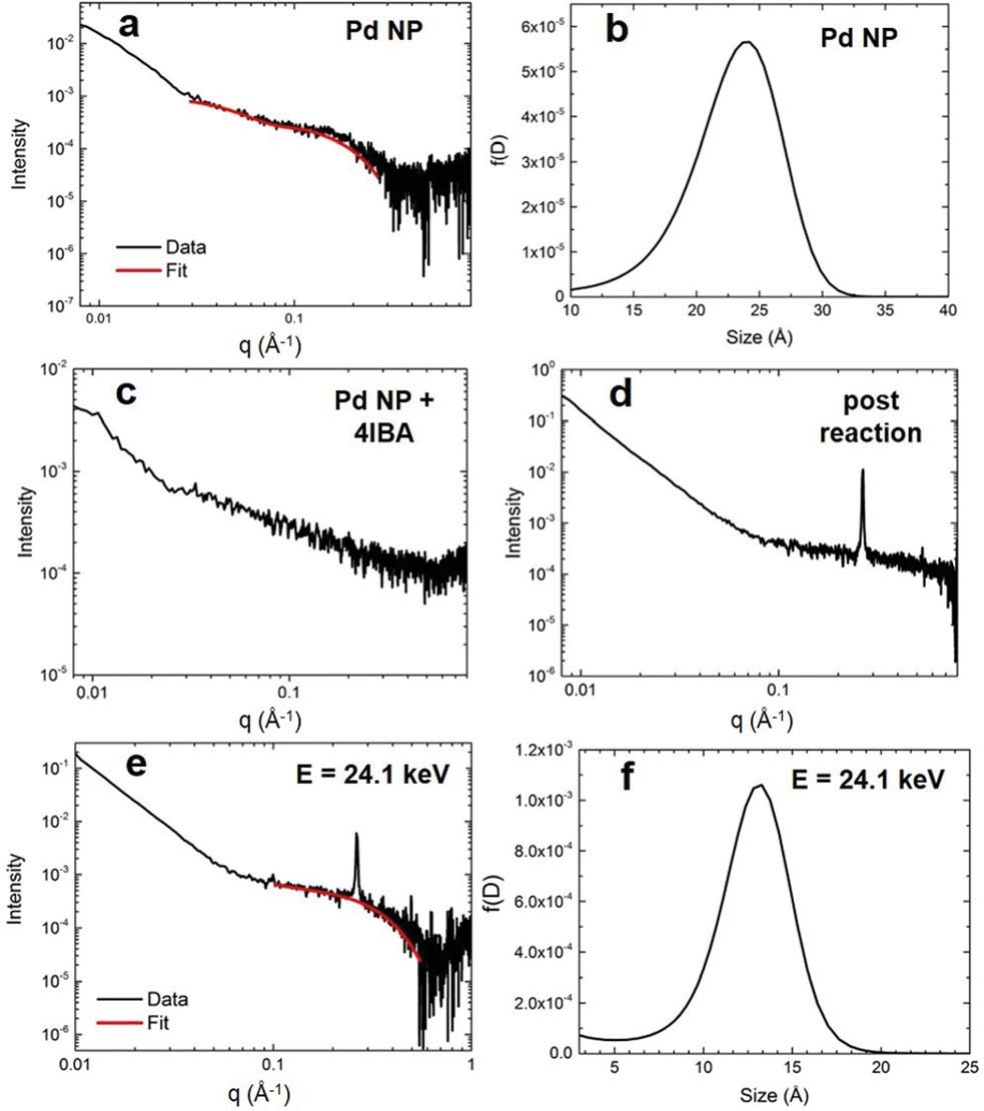
SAXS analysis. Part (a) shows the scattering data for the Pd4-capped Pd NPs in KOH before the reaction with (b) presenting the size distribution. Part (c) displays the scattering data for the Pd NPs in the presence of 4-IBA that isolates oxidative addition, while part (d) shows the scattering analysis for the materials post Stille coupling. Part (e) presents the ASAXS analysis of the NPs post Stille coupling at 24.1 keV, while (f) displays the corresponding size distribution.

To confirm the proposed reaction-based nanocatalyst changes *via* oxidative leaching observed by EXAFS, SAXS was employed to probe the Pd NP size. SAXS studies were conducted using the EXAFS experimental conditions, providing a direct comparison. The resulting scattering patterns were background subtracted, averaged, and subsequently analyzed using the Irena macros in Igor Pro.^33^ The pattern for the Pd4-capped Pd NPs in KOH displayed a broad feature at ∼0.2 Å^–1^, which can be modeled using a spherical form factor (Fig. 4a). From this, the size distribution of the NPs is centered at ∼24 Å (Fig. 4b), which is only slightly larger than the TEM determined size (2.0 ± 0.3 nm). When the Pd NPs were studied with the addition of the aryl halide that isolated the oxidative addition step, the SAXS pattern exhibited an absence of any tangible nanospherical scatters (Fig. 4c). Qualitatively, the NP feature is significantly broadened out, suggesting a complete loss of spherical materials. This is consistent with the EXAFS results that indicated a total loss of Pd–Pd bonds in the presence of 4-IBA due to oxidative-based Pd leaching.

When the Pd NPs were probed after the Stille coupling reaction was fully completed using SAXS, the pattern exhibited a diffraction peak at 0.264 Å^–1^ and a sharp power-law upturn at low *q* (Fig. 4d). The large diffraction peak corresponded to a *d*-spacing of 23.8 Å, likely from the crystalline reaction product that precipitated, while the power-law upturn at low *q* can be attributed to the sharp interfaces of the crystallized product. Given the substantial concentration differences between Pd and the Stille product (0.3 mol% Pd), a lack of Pd features in the pattern was not surprising. As such, anomalous SAXS (ASAXS) experiments were performed close to the Pd K-edge to increase the Pd contrast. The ASAXS pattern (Fig. 4e) still displays the same features of the SAXS pattern, but also exhibits a characteristic feature for spherical NPs at ∼0.3 Å^–1^. Modeling of this feature to a spherical form factor resulted in a size distribution centered at ∼13 Å (Fig. 4f), which is smaller than the initial Pd NPs. The decreased size supports the presence of smaller NPs, potentially resulting from the core of the original structures once the active disordered species have been leached.

**Scheme 1:**
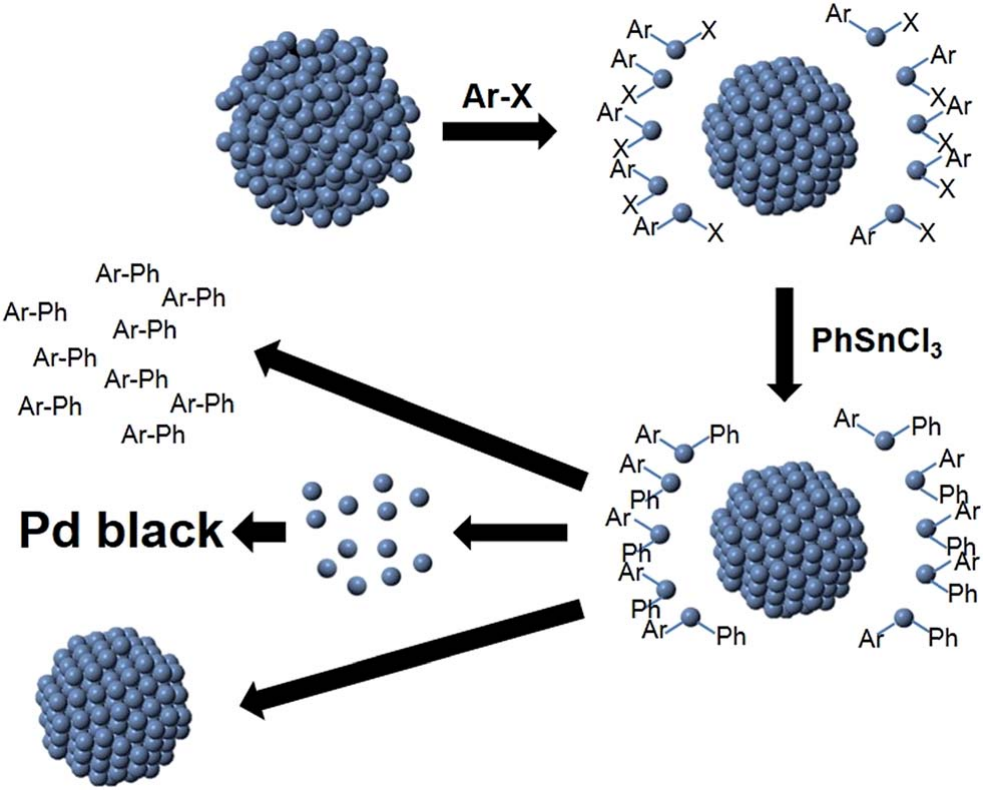
Pd atom leaching Stille coupling mechanism.

Based on both the EXAFS and SAXS results, a mechanism for Stille coupling catalyzed by peptide-capped Pd NPs is proposed, assuming that both the aryl halide and transmetalation components are present in the reaction mixture (Scheme 1). First, the Pd NPs are exposed to the aryl halide, which leaches the disordered Pd species (atoms or potentially small clusters) from the NP surface during oxidative addition, leaving behind a more ordered core. Next, transmetalation occurs, followed by reductive elimination, forming the product and releasing Pd^0^ atoms. These pre-leached Pd^0^ species are likely to be more reactive for oxidative addition than the Pd atoms on the more ordered core.^24^ Under this assumption, the remaining NP would become less reactive as compared to the abstracted Pd species in solution, suggesting that the pre-leached Pd components would continually cycle through the reaction. The smaller NP core is likely to be responsible for the contracted bond length, as observed by EXAFS, and the smaller diameter shown in SAXS. Upon reaction completion, the leached Pd atoms can either aggregate to Pd black, potentially form new NPs in the reaction medium, or deposit on the NP cores. Additional studies focused on the reductive elimination step are required to confirm the fate of the leached Pd species.

## Conclusions

In conclusion, X-ray-based studies have indicated that a leaching mechanism using peptide-capped Pd NPs for Stille coupling is occurring using unique aqueous and room temperature conditions. For this, highly disordered Pd atoms are leached from the NP surface during oxidative addition by the aryl halide. These leached metal species then drive Stille coupling in solution. Such detailed exploration of the mechanism of this reaction with NPs under sustainable conditions is important to understand the structure/function relationship of these materials. From such a mechanistic understanding using environmentally friendly conditions, the rational design of materials for optimized C–C coupling is possible. Such studies are presently underway in our labs. Furthermore, the X-ray-based approaches described here could be applied to other NP-driven reactions to elucidate structural/mechanistic effects, providing new routes for NP reaction analysis.

## Supplementary Material

Supplementary information
