# Supplementary material for: Atomic-scale identification of Pd leaching in nanoparticle catalyzed C–C coupling: effects of particle surface disorder
Source: Chem Sci. 2015 Jul 23;6(11):6413–9. doi: 10.1039/c5sc01424g (PMC6054123; doi:10.1039/c5sc01424g)
Supplement: Supplementary file 1 [file SC-006-C5SC01424G-s001.pdf]

Electronic Supplementary Material (ESI) for Chemical Science.
This journal is © The Royal Society of Chemistry 2015

Supporting Information

Atomic-Scale Identification of Pd Leaching in Nanoparticle Catalyzed C-C Coupling: Effects of Particle Surface Disorder

Beverly D. Briggs,[a,‡] Nicholas M. Bedford,[b,a,c‡,] Soenke Seifert,[d] Hilmar Koerner,[b] Hadi Ramezani-Dakhel,[e] H. Heinz,[e] Rajesh R. Naik,[b] Anatoly I. Frenkel,[f] and Marc R. Knecht[a*]

[a]Department of Chemistry, University of Miami, 1301 Memorial Drive, Coral Gables, FL 33146, United States

[b]Materials and Manufacturing Directorate, Air Force Research Laboratory, Wright-Patterson Air Force Base, Ohio 45433, United States

[c]Applied Chemicals and Materials Division, National Institute of Standards and Technology, 325 Broadway, MS 647, Boulder CO 80305, United States

[d]X-Ray Science Division, Argonne National Laboratory, 9700 S. Cass Ave, Argonne IL 60439, United States

[e] Department of Polymer Engineering, University of Akron, Ohio, 44325, United States

[f]Department of Physics, Yeshiva University, 245 Lexington Ave, New York, New York 10016, United States

Materials and Methods

Materials. K_2PdCl_4 and $PhSnCl_3$ were supplied from Sigma Aldrich. $NaBH_4$, *t*-butylphenol, *N*-methyl-*N*-(trimethylsilyl)-trifluoroacetamide (MSTFA), and $CDCl_3$ were purchased from Acros Organics. KOH, ethyl ether, *N,N*-dimethylformamide (DMF), dichloromethane (DCM), acetonitrile, methanol, and $NaSO_4$ were obtained from BDH Chemicals, while NaCl and HCl were from EMD Chemicals. 4-iodobenzoic acid was supplied from TCI America. Water used for all analyses was 18 MΩ•cm (Millipore, Ultrapure Synergy UV). Finally all peptide synthesizing materials including 9-fluoroenylmethoxycarbonyl (FMOC) protected amino acids and 4-hydroxymethylphenoxy acetyl (WANG) resins were purchased from Creosalus.

Methods

Peptide Synthesis. The Pd4 peptide (TSNAVHPTLRHL) was synthesized using standard solid phase peptide synthesis techniques on a TETRAS peptide synthesizer (Creosalus).[1] The crude peptide was then purified using reverse-phase HPLC with a water/acetonitrile gradient, and the molecular weight was confirmed with MALDI-TOF mass spectrometry.

Particle Synthesis. Pd4-capped Pd NPs were prepared based on a previously established protocol.[2] Briefly, 25 μL of 0.1 M K_2PdCl_4 was mixed with 100 μL of 10.0 mg/mL Pd4 peptide and 4.775 mL of water in a 20 mL glass vial. The mixture was allowed to complex on the benchtop for 30 min. Next, 100 μL of freshly made 0.1 M $NaBH_4$ was added, the vial was swirled to mix, and then the solution was allowed to reduce for 1 h prior to use.

Stille Coupling to Isolate Oxidative Addition. Stille coupling was conducted with an isolated oxidative addition step to probe its effects on the catalytic process. First, 620 mg of 4-IBA was dissolved in 40 mL of 2.25 M KOH. To this solution, 2.5 mL (0.05 mol%) of Pd NPs were added. The solution was allowed to stir for 30 min during which a loss of the characteristic brown Pd NP color was noted. After 30 min, 493 μL of $PhSnCl_3$ was added to the solution. From this point, 4 mL aliquots were taken at regular time intervals and quenched in 25 mL of 5% HCl. The aliquots were then extracted with three times with 30 mL of ethyl ether followed by a wash of two times with 20 mL of saturated NaCl. The organic layer was then dried over Na_2SO_4, after which 75 mg of *t*-butylphenol was added as an internal standard. The organic layer was then removed via rotary evaporation and analyzed via ^{1}H NMR and GC-MS.[2]

Standard Stille Coupling Reaction. When the materials were studied by EXAFS and SAXS after reaction completion, the catalytic process was studied that did not isolate oxidative addition. In this regard, identical reaction conditions and concentrations were used; however, the 4-IBA and $PhSnCl_3$ were commixed prior to addition of the Pd NPs. Upon reaction initiation via NP addition, characterization of the material followed using the standard approaches.

Characterization. ^{1}H NMR was conducted on a Bruker Advance Broadband 400 MHz NMR. ~1 mg of the solid sample was dissolved in 1 mL of $CDCl_3$ and then placed in an NMR tube. The integration of the peak at δ6.8 ppm, corresponding to the internal standard (*t*-butyl phenol), was compared to the integration of the peak at δ8.2 ppm, corresponding to the product, biphenylcarboxylic acid. GC-MS was conducted with an Agilent 6850 gas chromatograph (GC),

equipped with an Agilent 5975C mass spectrometer (MS). The samples were treated with MSTFA by stirring ~2 mg of the sample in 200 μL of the silane reagent for 2 h to produce trimethylsilyl ester analogues. The samples were then diluted in DCM to reach a concentration of ~1 mg/mL before analyzing.

EXAFS. EXAFS analysis was performed at Brookhaven National Laboratory (BNL) at the National Synchrotron Light Source (NSLS) on beamline X18B. The samples were prepared using 0.3 mol% Pd NP at 1.125 M KOH with standard Stille coupling conditions (0.5 mmol 4-IBA and 0.6 mmol $PhSnCl_3$) in a 4 mL total solution volume. The components of the solution were varied, as discussed above, depending on which step of the Stille coupling mechanism was being explored. These solutions were transferred to a kapton-window liquid cell. Energy scans were taken from –150 keV below to 50 keV above the Pd edge jump (24.353 keV). Fluorescence detectors were employed: either a Passivated Implanted Planar Silicon (PIPS) or 13-element Ge detector. Eight scans of each experimental condition were measured, averaged, and processed using the IFEFFIT software.[3]

SAXS. SAXS data were collected at the Advanced Photon Source (APS) at Argonne National Laboratory (ANL) on beamline 12-ID-C at 12 keV. The detector used was an in-house CCD detector. The samples were analyzed at 0.3 mol% Pd NP in 1.125 M KOH with standard Stille coupling conditions (0.5 mmol 4-IBA and/or 0.6 mmol $PhSnCl_3$) in 2 mL of total solution. Like the EXAFS studies, the components of the solution were varied depending on which step of the Stille coupling mechanism was being explored. The samples were transferred to 2 mm quartz capillaries for analysis. An exposure time of 0.1 s was used for 60 scans with a 40 s delay between each exposure. Spectra were background subtracted and averaged together using the Igor Pro software.[4]

Computational Analysis of Coordination Numbers. Coordination number were computed from molecular models of the peptide-covered nanoparticles using molecular dynamics simulations with the CHARMM-INTERFACE force field.[5] Atomically resolved structural models of the nanoparticles were obtained using data from High Energy XRD and pair distribution functions (PDFs) in experiment. The models were employed to examine the conformation and binding energy of peptides adsorbed on the surface in aqueous solution as well as the coordination numbers in equilibrium. The protocol to generate these structures and to carry out molecular dynamics simulations has been previously described in detail.[6]

In brief, the atomistic model for an average Pd nanoparticle derived by reductive synthesis in the presence of peptide Pd4 was obtained as follows. First, an atomistic model of bulk Pd (large supercell of the fcc unit cell) was processed into a model of a near-spherical nanoparticle with the desired average number of 319 atoms, which was derived from HR-TEM, XRD, and corresponding PDF data. The initial model was built by choosing an arbitrary center atom and a spherical cutoff at 10.5 Å, followed by deletion of surface atoms as needed. Then, PDFs were computed from the initial model and reverse Monte Carlo (RMC) simulations employed to alter the particle structure so that the computed PDF from the model particle fits closely to the experimental PDF. The final particle structure obtained from RMC was further relaxed in molecular dynamics simulation using the CHARMM-INTERFACE force field[5] to create a realistic surface structure; this step is necessary as the experimental HE-XRD and PDF data represent a size distribution over several particles while the entire particle size distribution is represented in a single nanoparticle model of average shape. The nanoparticle model was then

brought in contact with peptide Pd4 in solution to compute peptide binding conformations and the binding energy near monolayer coverage using several parallel replicas, advanced annealing and sampling protocols.[6] The same set of data was also previously obtained for various other nanoparticles derived from peptide mutants of the parent Pd4 peptide.

The computation of coordination numbers was carried out by an analysis of the number of nearest atomic neighbors in the equilibrium models, including Pd-Pd and Pd-C/O/N coordination. Thereby, neighbors within the first coordination shell of all Pd atoms were taken into account, *i.e.*, including Pd atoms in the core that only have Pd neighbors, as well as surface Pd atoms that have fewer Pd neighbors as well as some C, N, and O neighbors. The reported coordination number (CN) refers to the average number of Pd, C, N, or O neighbors per atom as an average over all atoms in the Pd nanoparticles, which is equivalent to the CN measured in XAFS studies. The analysis of the data shows an average Pd-Pd coordination number of 5.8 ± 0.2 for the Pd4-capped nanoparticles. The average Pd-N/O coordination number is 0.5 ± 0.1.

References

1. Chan, W. C.; White, P. D., *Fmoc Solid Phase Peptide Synthesis: A Practical Approach*. Oxford University Press: New York, NY, 2000.

2. Pacardo, D. B.; Sethi, M.; Jones, S. E.; Naik, R. R.; Knecht, M. R., Biomimetic Synthesis of Pd Nanocatalysts for the Stille Coupling Reaction. *ACS Nano* **2009,** *3* (5), 1288-1296.

3. Newville, M., IFEFFIT: Interactive XAFS Analyis and FEFF Fitting. *J. Synchrotron Rad.* **2001,** *8*, 322-324.

4. Ilavsky, J.; Jemian, P. R., Irena: Tool Suite for Modeling and Analysis of Small-Angle Scattering. *J. Appl. Cryst.* **2009,** *42*, 347-353.

5. (a) Heinz, H.; Lin, T.-J.; Mishra, R. K.; Emami, F. S., Thermodynamically Consistent Force Fields for the Assembly of Inorganic, Organic, and Biological Nanostructures: The INTERFACE Force Field. *Langmuir : the ACS journal of surfaces and colloids* **2013,** *29* (6), 1754-1765; (b) Heinz, H.; Vaia, R. A.; Farmer, B. L.; Naik, R. R., Accurate Simulation of Surfaces and Interfaces of Face-Centered Cubic Metals Using 12−6 and 9−6 Lennard-Jones Potentials. *J. Phys. Chem. C* **2008,** *112* (44), 17281-17290; (c) Ramezani-Dakhel, H.; Ruan, L. Y.; Huang, Y.; Heinz, H., Molecular Mechanism of Specific Recognition of Cubic Pt Nanocrystals by Peptides and the Concentration-Dependent Formation from Seed Crystals. *Adv. Funct. Mater.* **2015,** *25*, 1374-1384.

6. Bedford, N. M.; Ramezani-Dakhel, H.; Slocik, J. M.; Briggs, B. D.; Ren, Y.; Frenkel, A. I.; Petkov, V.; Heinz, H.; Naik, R. R.; Knecht, M. R., *submitted.*

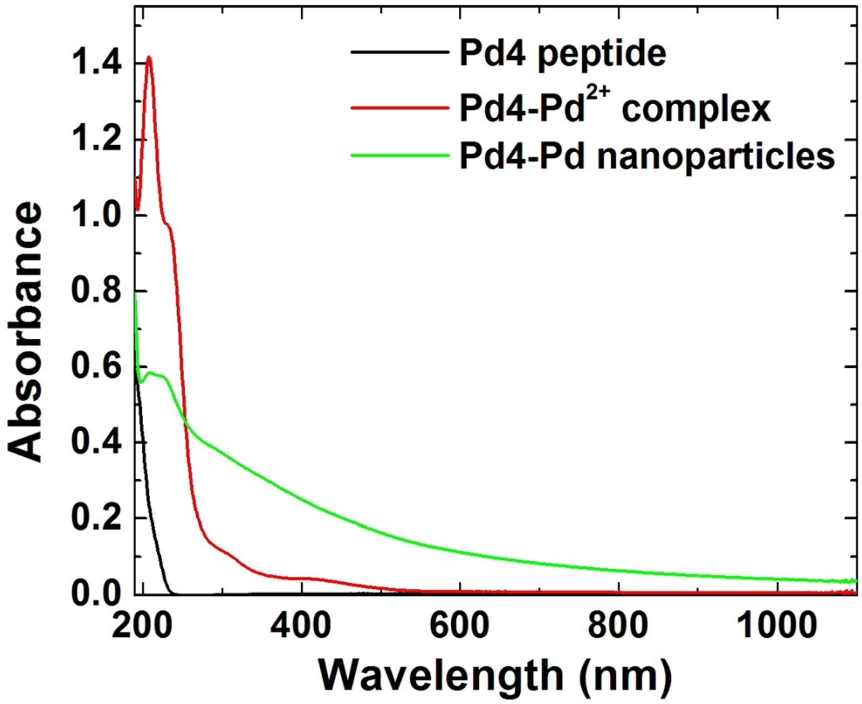

Figure S1. UV-vis analysis of the Pd4-capped Pd NPs before and after reduction.

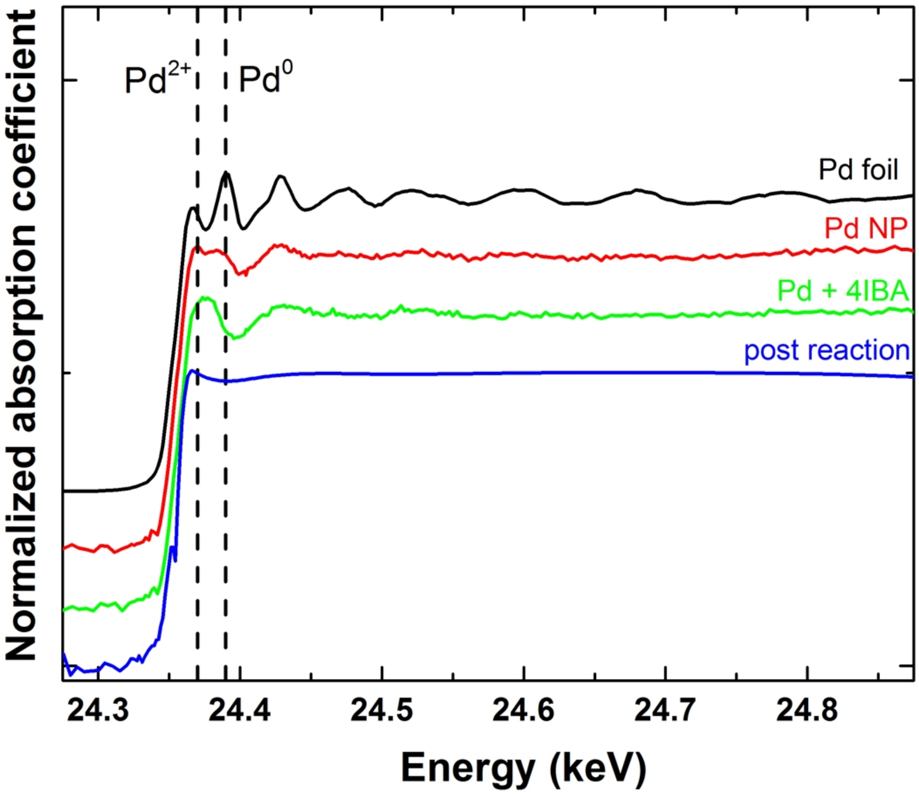

Figure S2. Raw EXAFS spectra used for transformation into r space.
